# Study on the Chemical Composition and Anti-Tumor Mechanisms of *Clausena lansium* Fruit By-Products: Based on LC-MS, Network Pharmacology Analysis, and Protein Target Validation

**DOI:** 10.3390/foods13233878

**Published:** 2024-11-30

**Authors:** Ziyue Zhang, Liangqian Zhang, Pengfei Wu, Yuan Tian, Yao Wen, Meina Xu, Peihao Xu, Ying Jiang, Nan Ma, Qi Wang, Wei Dai

**Affiliations:** 1 Teaching and Experimental Center, Guangdong Pharmaceutical University, Guangzhou 510006, China; ziyuezhang1103@126.com (Z.Z.);; 2 College of Pharmacy, Jinan University, Guangzhou 510632, Chinajiangyingjy0517@gmail.com (Y.J.); 3 Key Laboratory of Xinjiang Phytomedicine Resource and Utilization, Ministry of Education, School of Pharmacy, Shihezi University, Shihezi 832002, China; 4 School of Pharmacy, Guangdong Pharmaceutical University, Guangzhou 510006, China; 5 Comprehensive Experimental Teaching Center of Traditional Chinese Medicine, Yunfu Campus, Guangdong Pharmaceutical University, Yunfu 527500, China

**Keywords:** *Clausena lansium*, metabolomics, anti-tumor activity, network pharmacology, molecular docking

## Abstract

*Clausena lansium* (Lour.) Skeels, commonly known as Wampee, are valued for their edible and medicinal qualities, yet their pericarp and seeds are often discarded, resulting in wasted resources. This study investigates the anti-tumor potential of these by-products, focusing on their chemical composition and underlying mechanisms of action. A combination of metabolomics, network pharmacology, molecular docking, and experimental validation was employed in our study. Cytotoxicity screening demonstrated that the pericarp extract exhibited notable anti-tumor effects against MDA-MB-231 breast cancer cells, while the seed extract showed no similar activity. Chemical profiling identified 122 compounds in the pericarp and seeds, with only 26.23% overlap, suggesting that distinct compounds may drive the pericarp’s anti-tumor activity. Network pharmacology and molecular docking analyses identified PTGER3, DRD2, and ADORA2A as key targets, with several alkaloids, flavonoids, coumarins, and sesquiterpenes exhibiting strong binding affinities to these proteins. Western blot analysis further validated that the pericarp extract upregulated DRD2 and downregulated ADORA2A, indicating a possible mechanism for its anticancer effects. These findings suggest that Wampee pericarp holds promise as a source of active compounds with therapeutic potential for breast cancer, with implications for its use in the food and pharmaceutical industries.

## 1. Introduction

*Clausena lansium* (Lour.) Skeels, commonly known as Wampee, is a species from the genus *Clausena* in the Rutaceae family. Native to southern China, it is widely distributed in tropical and subtropical regions, including Vietnam and Malaysia [1]. The Wampee fruit is oval, with an approximate diameter of 2.0 cm. Both the pulp and the pericarp are edible and known for their high nutritional value and distinct flavor [2]. Previous studies have highlighted the presence of bioactive compounds in Wampee, such as phenolics, alkaloids, coumarins, and volatile oils [2,3,4], which exhibit a wide range of biological activities, including anti-inflammatory, antioxidant, neuroprotective, hepatoprotective, and anti-tumor effects [5,6,7,8,9]. Despite its known medicinal properties, the pericarp and seeds of Wampee fruit are often discarded during the processing of the fruit, leading to a significant loss of valuable resources. The growing recognition of the health benefits of Wampee fruit, along with the increasing interest in the sustainable use of plant by-products, has prompted the need for a more comprehensive investigation into the potential applications of these by-products, particularly their medicinal value.

Cancer remains a major global health challenge, imposing significant burdens on healthcare systems worldwide [10]. As a rich source of bioactive compounds, natural products have emerged as crucial resources in the development of anticancer therapies. These products not only serve as leads for the discovery of novel anti-tumor agents but also complement existing pharmacological treatments, offering new therapeutic targets for cancer intervention. Therefore, comprehensive research investigating the bioactivity, chemical composition, and mechanisms of natural products is essential for advancing cancer therapeutics [11,12]. Wampee fruit, widely regarded as safe for consumption, presents significant potential as a pharmaceutical supplement due to its rich array of bioactive compounds. Previous studies have primarily focused on the protective effects of Wampee on human health, examining its anti-inflammatory, antioxidant, and hepatoprotective properties [13,14,15,16]. While some research has reported the anti-tumor activity of Wampee, these findings have generally been limited to cytotoxicity assessments, and the mechanisms underlying its anticancer effects remain underexplored [8]. Given these gaps, further research is warranted to fully elucidate the anti-tumor potential and underlying mechanisms of Wampee fruit, particularly its by-products such as the pericarp and seeds.

In this study, we aim to investigate the anti-tumor activity of Wampee pericarp and seed by conducting cytotoxicity screening on various cancer cell lines. The chemical composition of the extracts from both pericarp and seeds was analyzed, revealing distinct differences in their constituent profiles. Network pharmacology and molecular docking techniques were employed to identify potential therapeutic targets for breast cancer, and experimental validation was conducted to assess the regulatory effects of pericarp extracts on these targets. By exploring the chemical composition and anti-tumor mechanisms of Wampee fruit by-products, this study aims to provide valuable insights into their potential therapeutic applications, particularly in the context of cancer management.

## 2. Materials and Methods

### 2.1. Chemicals and Reagents

Dulbecco’s modified eagle medium (DMEM) and streptomycin/penicillin were purchased from Thermo Fisher Scientific (Waltham, MA, USA) (Gibco, C11995500BT, 15140122, Seoul, Republic of Korea). Fetal Bovine Serum (FBS) was acquired from Excell Bio Company (Shanghai, China) (FCS500). BCA protein assay kits were bought from Beyotime Biotechnology (Shanghai, China) (P0009-1). PVDF membranes were obtained from MilliporeSigma (St. Louis, MO, USA) (IPVH00010). UPLC-Q-Orbitrap HRMS analysis was conducted using the Vanquish Flex UHPLC system and the Orbitrap Exploris 120 quadrupole electrostatic field orbital high-resolution mass spectrometer, both manufactured by Thermo Fisher Scientific (Waltham, MA, USA). WGL-23OB electric drying oven is produced in Tianjin Taisite Instrument (Tianjin, China) Co., Ltd. The DFY-300C Swing Crusher was sourced from Wenling Linda Machinery Co., Ltd. (Wenling, China). Precise weighing was performed using the ATY 1/24 million balance provided by Shimadzu Enterprise Management Co., Ltd. (Shanghai, China). The KQ-500DE Desktop CNC Ultrasonic Cleaner was obtained from Dongguan Keqiao Ultrasonic Equipment Co., Ltd. (Dongguan, China). Analytical-grade methanol was procured from Da Mao Chemical Reagent Co., Ltd., Tianjin, China, and chromatography-grade acetonitrile was acquired from Honeywell Trading (Shanghai) Co., Ltd., Shanghai, China. Distilled water from Watsons, Hong Kong, China, was utilized.

### 2.2. Plant Material and Extract Preparation

The pericarp and seeds of fresh Wampee were separated and put into the electric drying oven, respectively. The temperature was set to 55 °C, and the dried pericarp and seeds were obtained after 24 h. Then, the dried pericarp and seeds were made into powder by a DFY-300C Swing Crusher (Wenling Linda Machinery Co., Ltd., Wenling, China). Twenty grams of dried pericarp and seed powder from Wampee were separately combined with 500 mL of methanol and thoroughly mixed. The resulting mixtures underwent ultrasonic-assisted extraction at 50 °C for 30 min. After cooling to room temperature, the mixtures were centrifuged for 15 min at 1980× *g*. Subsequently, 200 μL of the supernatant was aliquoted and diluted with methanol to a final volume of 1 mL. The diluted solutions were meticulously mixed and then filtered through a 0.22 μm membrane filter for subsequent mass spectrometric analysis. The remaining supernatant was dried using a rotary evaporator to yield extracts.

### 2.3. Cell Culture

Human breast cancer cells (MDA-MB-231 and MCF-7), HEK-293T cells, and other cancer cells (HCT-116, A549, SW1990) were cultured in DMEM containing 10% FBS and 1% streptomycin/penicillin. Cells were incubated at 37 °C with 5% CO_2_ in a humidified atmosphere. When the cells reached 80–90% confluence, they were subcultured, and the medium was replaced every 2–3 days.

### 2.4. MTT Assay

The MTT (3-(4,5-dimethyl-2-thiazolyl)-2,5-diphenyl-2-H-tetrazolium bromide) assay was performed to evaluate cell proliferation. Cells were resuspended at a density of 5 × 10^4^ cells/mL, and 100 μL per well was seeded into a 96-well plate. Extracts from both pericarp and seeds at different concentrations were added and incubated at 37 °C for 48 h after an initial 24 h incubation at 37 °C;. Subsequently, 20 μL of MTT solution (5 mg/mL in medium) was added to each well. After a 4 h incubation, the culture medium was removed, and 100 μL of DMSO was added to dissolve the formazan. The plate was gently shaken in darkness for 20 min to ensure complete dissolution of the formazan. Absorbance at 490 nm was measured using BioTek microplate reader (EPOCH2NS-SN, Agilent Co., Ltd., Santa Clara, CA, USA).

### 2.5. LC-MS Analysis

UPLC-Q-Orbitrap HRMS analysis was performed using a Vanquish Flex UHPLC system coupled with an Orbitrap Exploris 120 quadrupole electrostatic field orbital mass spectrometer. Separation was achieved using a Hypersil GOLD C_18_ analytical column (100 mm × 2.1 mm, 5 µm) from Thermo Fisher Scientific, maintained at 35 °C. The mobile phase consisted of acetonitrile (A) and water/formic acid (0.1% *v*/*v*) (B), with gradient elution at a flow rate of 0.3 mL/min. The gradient conditions were as follows: 95% to 60% B over 0 to 15 min, 60% to 5% B over 15 to 25 min, and maintained at 5% B from 25 to 27 min. A sample injection volume of 2.0 µL was utilized.

The mass spectrometer operated in both positive and negative ion modes. The MS detection parameters were optimized with a spray voltage of +3.5 kV for positive ion mode and −2.8 kV for negative ion mode. The ion transfer tube temperature was set to 325 °C, with sheath gas, auxiliary gas, and sweep gas at 50, 8, and 1 Arb, respectively. The evaporator temperature was set to 350 °C, and the RF lens was adjusted to 70%. The scan range spanned from *m*/*z* 100 to 1500, with a resolution of 60,000 (MS) and 15,000 (MS2). Stepped normalized collision energy (NCE) settings of 20%, 40%, and 60% were applied. Orbitrap mass calibration was conducted weekly to ensure precise mass measurements.

### 2.6. LC-MS/MS Data Analysis

For data analysis, Compound Discoverer 3.3 software was employed. The raw data files were imported, and peak extraction and alignment were conducted using the compound identification method template. The secondary fragment spectra were matched against the mzCloud and mzVault databases. The matched results were filtered based on specific criteria, including the exclusion of blank background ions, ensuring a quality deviation of primary and secondary levels within 5 ppm, and requiring a minimum mzCloud or mzVault score of 80. The filtered ions were then compared with the compound information.

A molecular network was constructed using the online workflow available on the GNPS website. The precursor ion mass tolerance was set to 0.02 Da, with an MS/MS fragment ion tolerance of 0.02 Da. A network was generated, wherein edges were filtered to possess a cosine score exceeding 0.7 and comprise more than six matched peaks. Furthermore, edges connecting two nodes were retained in the network only if each node appeared in the respective top ten most similar nodes of the other. The matching of all network spectra and library spectra required a score surpassing 0.7, with a minimum of six matching peaks. The resultant data were downloaded and exported.

### 2.7. Network Pharmacology-Based Analysis

All identified compounds were converted to SMILES structures using the PubChem database (https://pubchem.ncbi.nlm.nih.gov/ (accessed on 28 November 2024)). The Swiss Target Prediction database was employed to retrieve potential targets for these compounds. Target genes related to breast cancer were obtained from the GeneCards database (www.genecards.org (accessed on 28 November 2024)). The potential genes associated with the pericarp of Wampee and the target genes of breast cancer were intersected and visualized using the Venny online tool (https://www.bioinformatics.com.cn/ (accessed on 28 November 2024)). Additionally, Metascape (https://metascape.org/ accessed on 28 November 2024)) was utilized to construct a protein–protein interaction (PPI) network and perform gene ontology (GO) enrichment and Kyoto Encyclopedia of Genes and Genomes (KEGG) enrichment analyses on Wampee pericarp-cancer targets. The online bioinformatics platform (https://www.bioinformatics.com.cn/ (accessed on 28 November 2024)) was used to visualize the top-ranked targets and obtain the differential expression of these targets between normal tissues and cancer tissues. The GEPIA database (http://gepia.cancer-pku.cn/ (accessed on 28 November 2024)) and HPA database (https://www.proteinatlas.org/ (accessed on 28 November 2024)) were subsequently used to acquire mRNA and protein expression levels of the core targets [17]. The mRNA expression level map was made by the GEPIA database. HPA database downloaded the sections of normal and BRCA.

### 2.8. Molecular Docking

Based on the selected optimal targets, the active compounds most related to these targets were identified, and molecular docking analysis was conducted to assess the binding ability of the active compounds to the key targets. The 3D structures of the selected ligands were obtained in SDF file format from PubChem (https://pubchem.ncbi.nlm.nih.gov/ (accessed on 28 November 2024)). OpenBabel-2.3.2 was used to convert the SDF files to MOL2 format. A subset of the 3D structures underwent MMFF94 minimization using ChemBio3D Ultra 14.0 and was saved as MOL2 files. Receptor proteins were sourced from the RCSB PDB database (https://www.rcsb.org/ (accessed on 28 November 2024)), with screening criteria set to Homo sapiens and a resolution of <3 Å, indicating higher resolution. In PyMOL, water and other extraneous atoms were removed from the proteins. The ligands underwent preprocessing in AutoDockTools 1.5.7, including adding hydrogens, computing Gasteiger charges, and assigning AD4 types, and were saved in PDBQT format. Similarly, small molecules were preprocessed in AutoDockTools 1.5.7, involving operations such as Choose Root, Detect Root, Show Root Expansion, and Choose Torsions, and were saved in PDBQT format. Subsequently, in AutoDockTools 1.5.7, the ligands and receptor proteins were loaded, and the receptor proteins were fully enveloped by the docking box. Semi-flexible docking was performed using Autogrid4, followed by molecular docking using Autodock4. Three favorable docking results were selected based on docking scores and visualized in 3D and 2D using PyMOL and LigPlot, respectively [18].

### 2.9. Western Blot Assay

Proteins were extracted from cells using RIPA lysis buffer containing a protease inhibitor cocktail and 0.1% Triton X-100. After centrifugation at 12,400× *g* rpm for 25 min at 4 °C, the supernatant was collected, and protein concentrations were measured using BCA protein assay kits according to the manufacturer’s instructions. Protein samples were separated by 10% SDS-PAGE and transferred to PVDF membranes. The membranes were blocked with 5% bovine serum albumin (BSA) at room temperature for 1 h and incubated with primary antibodies overnight at 4 °C. The primary antibodies used were against ADORA2A (ABclonal, A1587, ABclonal Biotechnology Co., Ltd., Wuhan, China), DRD2 (Bioss, bs-1008R, Bioss Biotechnology Co., Ltd., Beijing, China), PTGER3 (Bioss, bs-1876R, Bioss Biotechnology Co., Ltd., Beijing, China), β-actin (Abcam, ab8227, Abcam pl, Cambridge, UK), and α-tubulin (Abcam, ab4074). The membranes were then incubated with HRP-conjugated goat-anti-rabbit secondary antibodies (Cell Signaling Technology, 7074S, Cell Signaling Technology, Inc., Danvers, MA, USA) at room temperature for 2 h. Signal intensity was detected using West Pico Plus (Thermo, 34577, Thermo Fisher Scientific, Waltham, MA, USA) on a chemiluminescence imaging system (Cytiva, Amersham ImageQuant 800, Cytiva, Uppsala, Sweden) and quantified using ImageJ 1.53k software.

### 2.10. Statistical Analysis

Statistical analysis was conducted using GraphPad Prism 8 software (San Diego, CA, USA). Multiple comparisons between experimental groups were evaluated using one-way ANOVA. Data were presented as mean ± standard deviation (SD). A significance level of *p* < 0.05 was considered statistically significant.

## 3. Results and Discussion

### 3.1. Evaluation of Anticancer Properties for Pericarp Extract and Seed Extract

**Figure 1:**
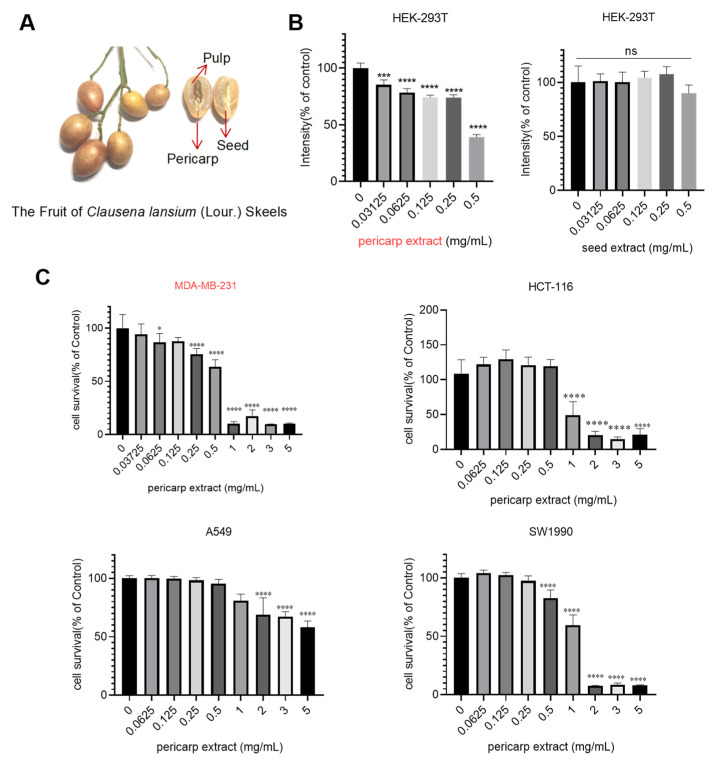
Cytotoxicity evaluation of pericarp extract and seed extract. (**A**) Image of Wampee pericarp and seed. (**B**) Cells were treated with different concentrations of pericarp extract and seed extract, and cell viability was assessed using the MTT assay after 48 h. (**C**) Cytotoxic effects of pericarp extract on MDA-MB-231, HCT-116, A549, and SW1990 cell lines. The cell viability was assessed after 48 h of exposure to varying concentrations of pericarp extract using the MTT assay. Results are expressed as a percentage of the control, and they represent the mean ± SD of more than three independent experiments. Ns: no significance, * *p* < 0.05, *** *p* < 0.001 and **** *p* < 0.0001.

Fresh Wampee fruits were harvested, and the peel, along with the seed, was separated for the preparation of crude extracts (Figure 1A). Then, we evaluated the cytotoxic effects of those extracts using HEK-293T cells as a model system. The results, depicted in Figure 1B, revealed that pericarp extract exhibited significant cytotoxic activity against HEK-293T cells at a concentration of 0.4513 mg/mL. In contrast, seed extract did not significantly affect cell proliferation. Based on these findings, we selected pericarp extract for further detailed analysis. The cytotoxic effects of pericarp extract were investigated on various tumor cell lines, including the MDA-MB-231 human breast cancer cell line, HCT-116 colon cancer cell line, A549 non-small cell lung cancer cell line, and SW1990 pancreatic cancer cell line. As shown in Figure 1C, the result demonstrated that pericarp extract exhibited significant cytotoxicity across all tested tumor cell types. Notably, pericarp extract displayed heightened sensitivity in exerting cytotoxic effects on the MDA-MB-231 cell. When the processing concentration is 0.25 mg/mL, the pericarp extract exhibits notable inhibitory effects on breast cancer cells. These findings prompted further investigation into the mechanisms by which pericarp extract inhibits the growth of breast cancer cells.

### 3.2. LC-MS Identification of Phytochemicals in the Pericarp and Seeds of Wampee

**Figure 2:**
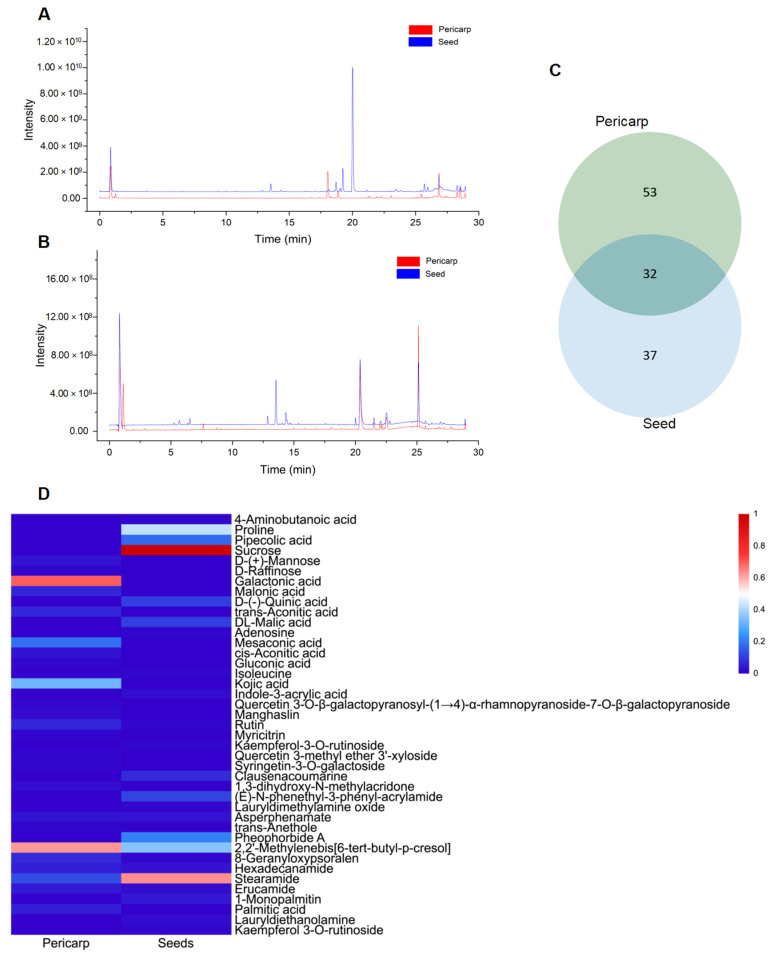
Mass spectrometry analysis of skin and seed metabolites of Wampee. (**A**) Mass spectrometry analysis of Wampee pericarp and seed metabolites in positive ion mode. (**B**) Mass spectrometry analysis of Wampee pericarp and seed metabolites in negative ion mode. (**C**) Analysis of pericarp and seed metabolites of Wampee. (**D**) Heatmap of pericarp and seed metabolites in Wampee, representing compounds with a peak area ratio greater than 1% relative to the highest component.

The observed differences in cytotoxicity between fruit pericarp extract and seed extract can be attributed to variations in their chemical compositions. Consequently, identifying the distinct compounds present in each extract may enhance the anti-tumor activity of pericarp extracts. In light of this, a comprehensive analysis of the phytochemical constituents in Wampee’s pericarp and seeds was conducted using UPLC-Q-Orbitrap-MS technology. The analytical process, supported by Compound Discoverer 3.3, GNPS database consultation, and existing literature, identified 122 compounds across the pericarp and seeds. These compounds include 39 alkaloids, 23 flavonoids, 19 organic acids, 7 coumarins, 8 esters, 6 phenols, 4 phenylpropanoids, 5 sugars, 2 ketones, 5 terpenoids, 2 ethers, 1 alcohol, and 1 aldehyde. Detailed compound information is presented in Tables S1 and S2, and the ion current diagrams are shown in Figure 2A,B. A comparative analysis revealed 85 compounds in the pericarp, with alkaloids (20) and flavonoids (20) being notably prevalent, along with organic acids (13), esters (7), and a miscellaneous group of 25 other types. In contrast, the seeds contained 69 compounds, primarily alkaloids (31), followed by organic acids (12), flavonoids (7), coumarins (4), and a group of 15 other types. This comparison highlights a shared chemical signature of 32 compounds across both tissues, including a significant overlap in alkaloids (13) and organic acids (6), suggesting a potential common biosynthetic pathway or functional role. The shared compounds also include flavonoids (4), esters (3), sugars (2), alcohol, phenol, coumarin, and a terpenoid, indicating a complex interplay of biochemical processes within Wampee.

The pericarp’s chemical profile is characterized by a substantial presence of both alkaloids and flavonoids, known for their diverse biological activities, including antioxidant, antimicrobial, and, notably, anti-tumor properties. The seeds, however, exhibit a more pronounced concentration of alkaloids, often associated with the plant’s defense mechanisms and contributing to its pharmacological profile. A total of 32 shared compounds, accounting for approximately 26.23% of the total identified components (as shown in Figure 2C), suggest a degree of biochemical unity between the pericarp and seeds. However, as indicated in Figure 2D, there are significant differences in the major metabolites of the pericarp and seeds of Wampee, with the distinct distribution of certain compounds highlighting their specific functional roles. This comparative metabolite analysis not only enhances our understanding of the chemical complexity of Wampee fruit but also lays the foundation for further exploration into its potential applications in pharmacology and nutrition.

### 3.3. The Fragmentation Pattern of the Identified Compounds

The mass spectrometry fragmentation patterns obtained through collision-induced dissociation (CID) provide accurate identification of compound structures and offer insights into their potential cleavage pathways. This approach serves as a valuable reference for the mass spectrometric identification of compounds in the Wampee pericarp. By analyzing the fragmentation behavior of key compounds, we can identify characteristic fragment ions that not only assist in structural elucidation but also provide molecular signatures for differentiating various compounds. This detailed analysis of fragmentation pathways, particularly for alkaloids, flavonoids, and coumarins, will lay the foundation for further structural analysis and functional studies of the chemical constituents in Wampee.

#### 3.3.1. Alkaloids Compounds

**Figure 3:**
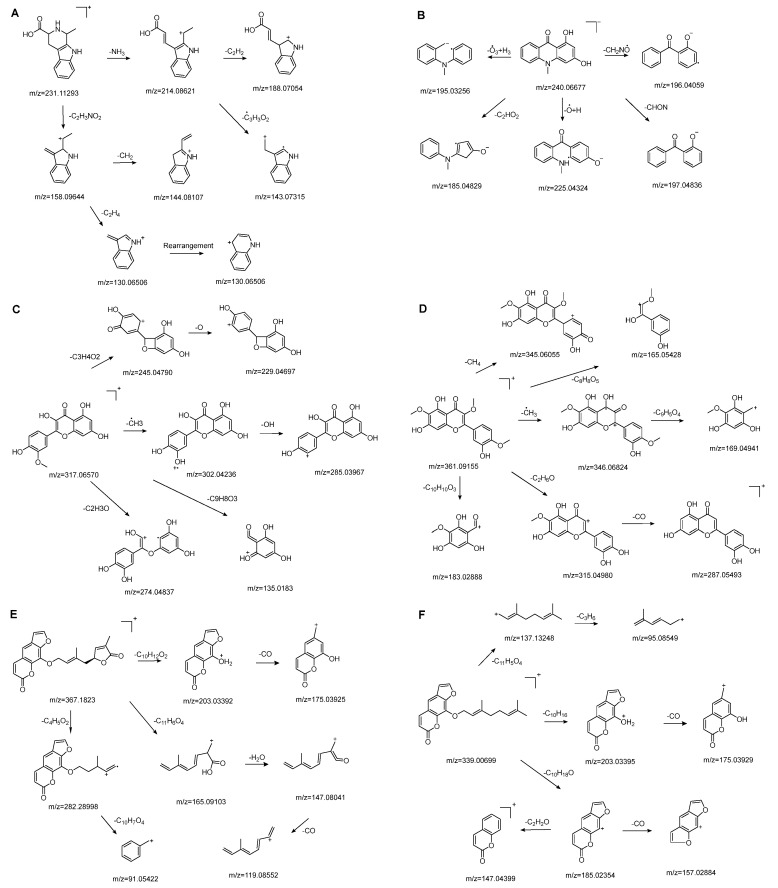
Possible fragmentation pathways. (**A**) 1,2,3,4-Tetrahydro-3-carboxyharmane; (**B**) 1,3-dihydroxy-N-methylacridone; (**C**) Isorhamnetin; (**D**) Centaureidin; (**E**) Wampetin; (**F**) 8-geranyloxypsoralen.

Alkaloids are one of the main classes of compounds found in Wampee. A total of 39 alkaloids were identified in the pericarp and seeds, with nitrogen-containing heterocyclic compounds constituting a significant portion. The quasi-molecular ion peak of compound 38 was observed at *m*/*z* 231.11293 [M+H]^+^, corresponding to the molecular formula C_13_H_14_N_2_O_2_, as determined using Xcalibur 4.0 software. Various fragment ions were formed through collision-induced dissociation. The primary fragmentation pathway likely involves the cleavage of the nitrogen-containing six-membered ring. One pathway produces *m*/*z* 214.08621 ([M+H−NH_3_]^+^), followed by *m*/*z* 188.07054 ([M+H−NH_3_−C_2_H_2_]^+^) and *m*/*z* 143.07315 ([M+H−NH_3_−C_3_H_3_O_2_]^+^). Another pathway yields *m*/*z* 158.09644 ([M+H−C_2_H_3_NO_2_]^+^), *m*/*z* 144.08107 ([M+H−C_2_H_3_NO_2_−CH_2_]^+^), and *m*/*z* 130.06506 ([M+H−C_2_H_3_NO_2_−C_2_H_4_]^+^). The fragment ion at *m*/*z* 130.06506 may rearrange to form a more stable six-membered heterocyclic structure. Based on the literature [19], the quasi-molecular ion peak of 1,2,3,4-Tetrahydro-3-carboxyharmane in positive ion mode was observed at *m*/*z* 214, with secondary fragments at *m*/*z* 214, 170, and 158, consistent with the observed data. Therefore, compound 38 was identified as 1,2,3,4-Tetrahydro-3-carboxyharmane. The fragment ion at *m*/*z* 130.06506, due to its potential stability from rearrangement, may serve as a characteristic ion for identifying this compound. The proposed mass fragmentation pathways are illustrated in Figure 3A.

For compound 67, the quasi-molecular ion peak was detected at *m*/*z* 240.06677 [M−H]^−^, with a molecular formula of C_14_H_11_NO_3_, as fitted by Xcalibur 4.0 software. Upon collision-induced dissociation, various fragment ions were generated. The primary fragmentation pathway involves the cleavage of the nitrogen-containing six-membered ring and the loss of the hydroxyl group from the benzene ring, resulting in *m*/*z* 195.03256 ([M−H−O_3_+H_3_]^−^) and *m*/*z* 196.04059 ([M−H−CH_2_NO]^−^). Other significant fragment ions include *m*/*z* 225.04324 ([M−H−O+H]^−^) and *m*/*z* 197.04836 ([M−H−CHON]^−^). A secondary pathway involves the rearrangement of the benzene ring, following the cleavage of the heterocyclic ring, to form a five-membered ring, yielding *m*/*z* 185.04829 ([M−H−C_2_HO_2_]^−^). Comparing these results with the reference [20], the data are consistent with the known compound 1,3-dihydroxy-N-methylacridone. The proposed mass fragmentation pathways are depicted in Figure 3B.

#### 3.3.2. Flavonoid Compounds

Flavonoids are one of the main classes of compounds found in Wampee, with 23 flavonoids identified. The fragmentation of flavonoids often occurs through the Retro-Diels-Alder (RDA) reaction on the C ring or by the loss of neutral small molecules or groups, such as H_2_O, CH_3_, and CO, producing various secondary fragments. The quasi-molecular ion peak of compound 59 was detected at *m*/*z* 317.06570 [M+H]^+^, corresponding to the molecular formula C_16_H_12_O_7_, as determined by Xcalibur 4.0 software. Upon collision-induced dissociation, several fragment ions were formed. The main fragmentation pathways are as follows: First, the methoxy group on the B ring detaches, resulting in *m*/*z* 302.04236 ([M+H−CH_3_]^+^) and *m*/*z* 285.03967 ([M+H−CH_3_−OH]^+^). Second, the RDA reaction occurs on the C ring, leading to ring opening at different bond positions and forming distinct secondary fragments, such as *m*/*z* 245.04790 ([M+H−C_3_H_4_O_2_]^+^), *m*/*z* 229.04697 ([M+H−C_3_H_4_O_2_−O]^+^), *m*/*z* 274.04837 ([M+H−C_2_H_3_O]^+^), and *m*/*z* 135.0183 ([M+H−C_9_H_8_O_3_]^+^). According to the literature [21], the quasi-molecular ion peak of Isorhamnetin appears at *m*/*z* 317, with secondary fragments at *m*/*z* 302 and 257 in the positive ion mode, consistent with the observed data. Thus, compound 59 was identified as Isorhamnetin. Additionally, the secondary fragments generated at different positions of the C ring, such as *m*/*z* 274.04837 and *m*/*z* 245.04790, may serve as characteristic ions for identifying this compound. The proposed mass fragmentation pathways are illustrated in Figure 3C.

For compound 65, the quasi-molecular ion peak was observed at *m*/*z* 361.09155 [M+H]^+^, corresponding to the molecular formula C_18_H_16_O_8_, as determined by Xcalibur 4.0 software. The main fragmentation pathways include the loss of methoxy groups at different positions on the flavonoid aglycone, leading to the formation of *m*/*z* 346.04824 ([M+H−CH_3_]^+^), *m*/*z* 345.06055 ([M+H−CH_4_]^+^), and *m*/*z* 315.04980 ([M+H−C_2_H_6_O]^+^), as well as *m*/*z* 287.05493 ([M+H−C_2_H_6_O−CO]^+^). Additionally, the RDA ring-opening reaction on the C ring results in the formation of various secondary fragments, including *m*/*z* 165.05428 ([M+H−C_8_H_8_O_5_]^+^), *m*/*z* 169.04941 ([M+H−CH_3_−C_9_H_5_O_4_]^+^), and *m*/*z* 183.02888 ([M+H−C_10_H_10_O_3_]^+^). According to the reference [22], the quasi-molecular ion peak of Centaureidin was observed at *m*/*z* 361.091, with secondary fragments at *m*/*z* 345.063, 328.058, 303.051, and 285.039, consistent with the identified compound. Furthermore, the secondary fragments generated at different positions of the C ring, such as *m*/*z* 165.05428 and *m*/*z* 183.02888, may serve as characteristic ions for identifying this compound. The proposed mass fragmentation pathways are depicted in Figure 3D.

#### 3.3.3. Coumarins Compounds

The quasi-molecular ion peak of compound 104 was detected at *m*/*z* 367.18230 [M+H]^+^, with a molecular formula of C_21_H_18_O_6_, as determined by Xcalibur 4.0 software. Different fragment ions were formed through collision-induced dissociation. The main fragmentation pathways include the breaking of the side chains of the parent nucleus to varying degrees, resulting in fragments at *m*/*z* 203.03392 ([M+H−C_10_H_12_O_2_]^+^) and *m*/*z* 282.28998 ([M+H−C_4_H_5_O_2_]^+^). Further fragmentation of the parent nucleus or side chain, particularly involving the cleavage of the oxygen-containing five-membered ring, leads to the formation of *m*/*z* 175.03925 ([M+H−C_10_H_12_O_2_−CO]^+^), *m*/*z* 165.09103 ([M+H−C_11_H_6_O_4_]^+^), *m*/*z* 147.08041 ([M+H−C_11_H_6_O_4_−H_2_O]^+^), *m*/*z* 119.08552 ([M+H−C_11_H_6_O_4_−H_2_O−CO]^+^), and *m*/*z* 91.05422 ([M+H−C_4_H_5_O_2_−C_10_H_7_O_4_]^+^). Comparing these results with the literature [23], the simulated molecular weight matched that of the compound, and given its higher concentration in Wampee, it was inferred that the compound is Wampetin. Additionally, we observed that this compound tends to break at the carbon-oxygen bond of the side chain, producing secondary fragments at *m*/*z* 203.03392 or *m*/*z* 282.28998, which may serve as characteristic ions for this compound, aiding in its subsequent identification. The proposed mass fragmentation pathways are illustrated in Figure 3E.

Compound 89 exhibited a quasi-molecular ion peak at *m*/*z* 339.00699 [M+H]^+^, with a molecular formula of C_21_H_22_O_4_, as fitted by Xcalibur 4.0 software. Various fragment ions were generated upon collision-induced dissociation. The main fragmentation pathways involve the cleavage of carbon-oxygen bonds of the oxygen atoms in the side chain of the parent nucleus, forming *m*/*z* 203.03395 ([M+H−C_10_H_15_]^+^) and *m*/*z* 185.02354 ([M+H−C_10_H_18_O]^+^). Further fragmentation of the parent nucleus or side chain, particularly the cleavage of the oxygen-containing five-membered or lactone ring, results in the formation of *m*/*z* 175.03929 ([M+H−C_10_H_15_−CO]^+^), *m*/*z* 165.09103 ([M+H−C_11_H_6_O_4_]^+^), *m*/*z* 147.04399 ([M+H−C_10_H_18_O−C_2_H_2_O]^+^), *m*/*z* 157.02884 ([M+H−C_10_H_18_O−CO]^+^), *m*/*z* 137.13248 ([M+H−C_11_H_5_O_4_]^+^), and *m*/*z* 95.08549 ([M+H−C_11_H_5_O_4_−C_3_H_6_]^+^). According to the literature [24], the quasi-molecular ion peak of 8-geranyloxypsoralen in positive ion mode was observed at *m*/*z* 339, with secondary fragments at *m*/*z* 203 and 147, consistent with the identified compound. Thus, compound 89 was inferred to be 8-geranyloxypsoralen. Additionally, the fragmentation patterns of compounds 89 and 104 suggest that the presence of furanocoumarin structures in these compounds results in characteristic secondary fragments at *m*/*z* 203 and 147. The cleavage pathway indicates that the side chain breaks to form a furanocoumarin nucleus (*m*/*z* 203) and further breaks to form fragments (*m*/*z* 147). These characteristic ions provide valuable reference points for the identification of furanocoumarin compounds. The proposed mass fragmentation pathways are depicted in Figure 3F.

### 3.4. Network Pharmacology Analysis

#### 3.4.1. Target Screening of the Pericarp of Wampee and Breast Cancer

**Figure 4:**
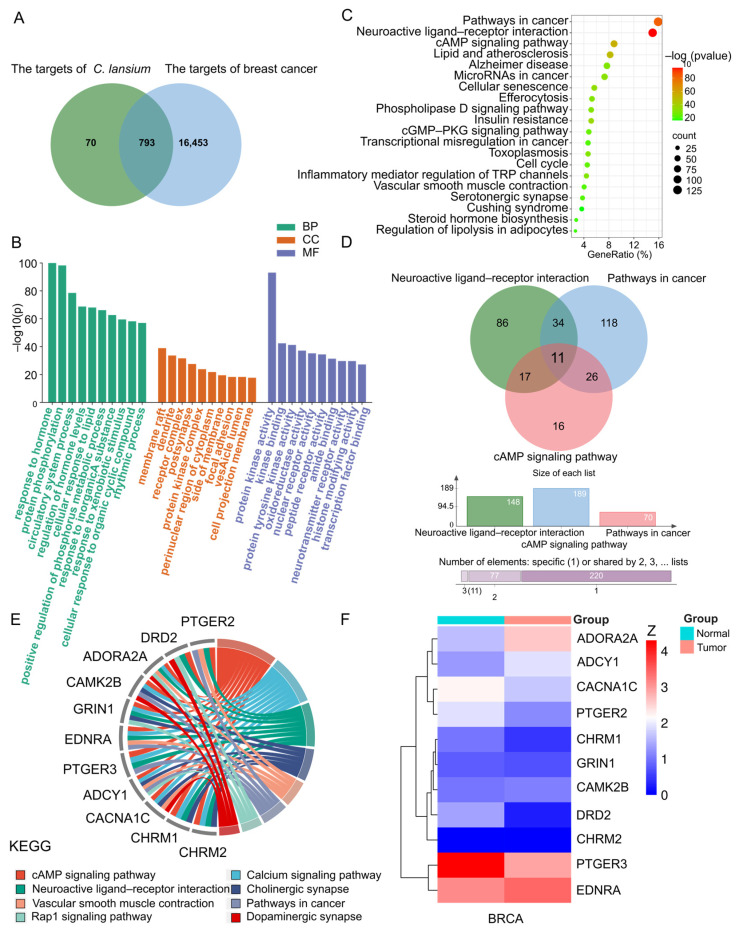
Network pharmacology analysis. (**A**) Venn diagram showing the overlapping targets between the pericarp of Wampee and breast cancer. (**B**) GO enrichment analysis, with the top 10 terms listed. (**C**) KEGG pathway enrichment analysis of key targets, listing the top 20 pathways. (**D**) Venn diagram of the intersection of target genes from the three core enrichment pathways. (**E**) Circos plot depicting the enrichment of core target genes. (**F**) Clustered heat map of the overlapping core target genes.

Given the significant inhibitory effects of Wampee pericarp on human breast cancer cells, we conducted a network pharmacology analysis. Using Swiss Target Prediction, we initially predicted 86 potential drug targets, which expanded to 863 after removing duplicates. Breast cancer-related targets were then queried from the GeneCards library, resulting in a comprehensive list of 17,246 targets associated with breast cancer. By comparing the targets of Wampee pericarp with the identified breast cancer-related targets using a Venn diagram, we identified 793 shared targets (Figure 4A). These shared targets represent potential candidates for breast cancer treatment by Wampee pericarp.

#### 3.4.2. GO and KEGG Enrichment Analysis of Key Targets

GO enrichment and KEGG enrichment analyses were performed on the 793 potential therapeutic target genes using the Metascape database. GO enrichment analysis covered MF, BP, and CC. In BP enrichment analysis, the target genes showed significant enrichment in responses to hormones, protein phosphorylation, and circulatory system processes. In CC enrichment analysis, the target genes were notably enriched in membrane rafts, dendrites, and receptor complexes. For MF enrichment analysis, the target genes were significantly enriched in protein kinase activity, kinase binding, and protein tyrosine kinase activity. The GO enrichment diagram is presented in Figure 4B.

KEGG enrichment analysis revealed that these potential therapeutic target genes were primarily enriched in the Pathways in Cancer, Neuroactive Ligand–Receptor Interaction, and cAMP Signaling Pathway. This pattern suggests involvement in critical pathways associated with cancer progression and signal modulation. The KEGG enrichment bubble diagram illustrating these findings is shown in Figure 4C.

#### 3.4.3. Target-Pathway Network and Analysis

A topological attribute analysis of the KEGG enrichment results indicated that “Pathways in cancer,” “Neuroactive ligand–receptor interaction,” and the “cAMP signaling pathway” had the highest degree of enrichment, with the “Neuroactive ligand–receptor interaction” pathway showing the highest degree of enrichment. A Venn diagram visualizing the target genes associated with these three pathways revealed 11 common core genes: PTGER2, DRD2, ADORA2A, CAMK2B, GRIN1, EDNRA, PTGER3, ADCY1, CACNA1C, CHRM1, and CHRM2 (Figure 4D). Subsequent enrichment analysis of these core target genes highlighted a series of cancer-related pathways, further corroborating our findings (Figure 4E).

**Figure 5:**
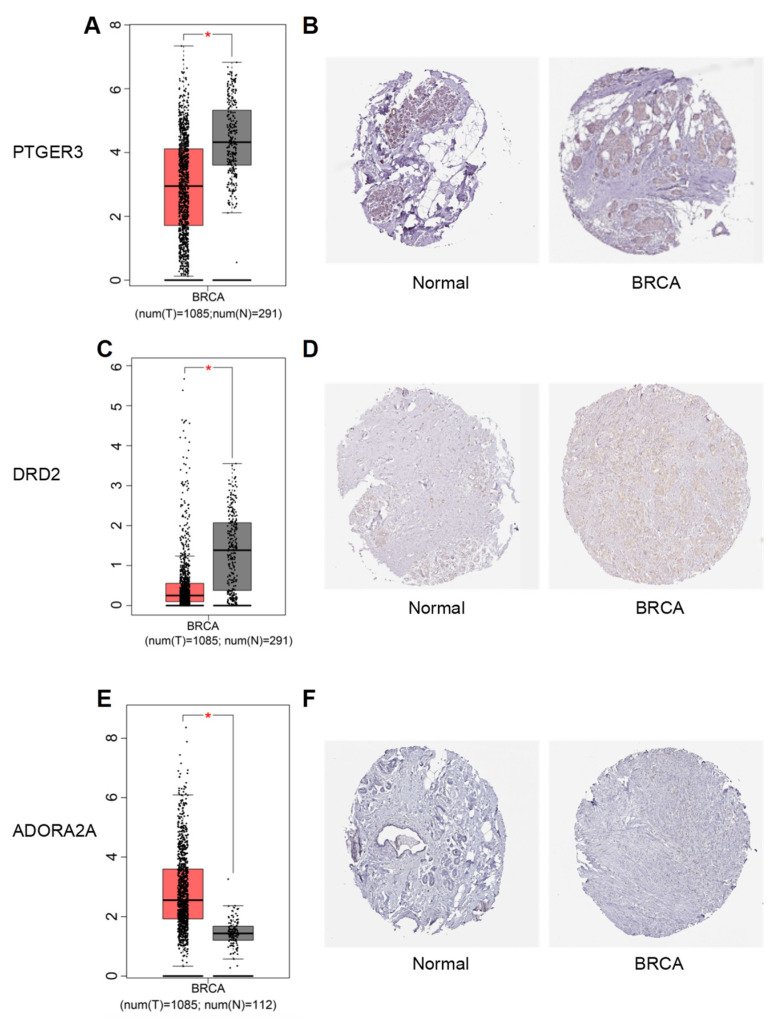
Expression levels of the core target genes. Panels (**A**,**C**,**E**) show the mRNA expression levels of three core genes in the TCGA cancer dataset. Panels (**B**,**D**,**F**) depict the protein expression levels of the same three core genes in clinical specimens from the HPA database. Note: BRCA refers to breast invasive carcinoma. *: This indicates that the expression matches the normal data of TCGA cancer dataset.

Furthermore, the GEPIA and HPA databases were utilized to investigate the mRNA and protein expression levels of core genes in normal and tumor tissues. The results identified PTGER3, DRD2, and ADORA2A as the most differentially expressed genes (Figure 4F). Among these, PTGER3 and DRD2 showed downregulated mRNA and protein expression in breast cancer tissues, while ADORA2A displayed upregulated expression compared to normal tissues (Figure 5).

### 3.5. Analysis of Molecular Docking Results

Molecular docking simulation is a powerful tool for exploring the optimal binding modes between protein receptors and small molecule active compounds. In this study, molecular docking was performed between the key active compounds identified through network pharmacology analysis (compound information is provided in Table 1) and the screened target proteins. Lower binding energy values indicate more stable interactions between the protein receptor and the small molecule active compound. Generally, binding energies below −5 kcal/mol suggest good binding affinity between the protein and ligand [25].

As shown in Table 2, among the seven compounds that demonstrated good binding affinity with the target proteins, two are coumarin derivatives (Wampetin and 2′,3′-Epoxyindicolactone), two are alkaloids (1,3-dihydroxy-N-methylacridone and 1,2,3,4-Tetrahydro-3-carboxyharmane), two are sesquiterpenes (Nootkatone and (+)-ar-Turmerone), and one is a flavonoid (Myricetin). Notably, 1,3-dihydroxy-N-methylacridone exhibited strong binding capabilities with both DRD2 and ADORA2A. The docking score of Wampetin with protein 6m9t was −6.19 kcal/mol, while the docking scores of Nootkatone, 1,3-dihydroxy-N-methylacridone, (+)-ar-Turmerone, and 1,2,3,4-Tetrahydro-3-carboxyharmane with protein 7jvr were −7.56 kcal/mol, −6.99 kcal/mol, −5.85 kcal/mol, and −5.73 kcal/mol, respectively. Additionally, the docking scores of Myricetin, 1,3-dihydroxy-N-methylacridone, and 2′,3′-Epoxyindicolactone with protein 5iu4 were −5.7 kcal/mol, −5.5 kcal/mol, and −5.19 kcal/mol, respectively.

**Figure 6:**
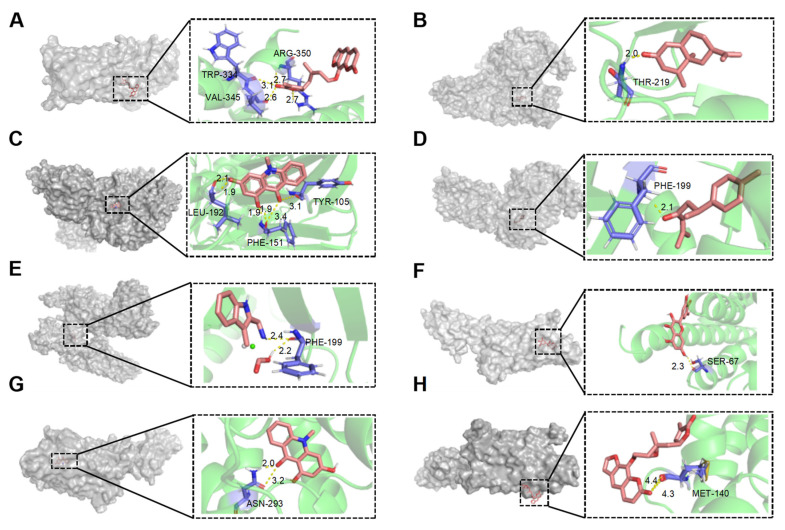
Molecular docking interactions of various compounds with target proteins. The panels illustrate the binding interactions for different compounds. (**A**) Wampetin with protein 6m9t; (**B**) Nootkatone with protein 7jvr; (**C**) 1,3-Dihydroxy-N-methylacridone with protein 7jvr; (**D**) (+)-ar-Turmerone with protein 7jvr; (**E**) 1,2,3,4-Tetrahydro-3-carboxyharmane with protein 7jvr; (**F**) Myricetin with protein 5iu4; (**G**) 1,3-Dihydroxy-N-methylacridone with protein 5iu4; (**H**) 2′,3′-Epoxyindicolactone with protein 5iu4. Each figure presents a 3D macroscopic view of the docking result on the left and a 3D interaction view on the right, with red representing the small molecule compound ligand and blue indicating the amino acid residues.

Figure 6A–H illustrate the primary types of interactions between receptor proteins and small molecule active compounds, including hydrogen bonds and van der Waals forces. For instance, Wampetin formed four hydrogen bonds with amino acid residues TRP-344, VAL-345, and ARG-350 of protein 6m9t. Nootkatone formed one hydrogen bond with Thr-219 of protein 7jvr. 1,3-dihydroxy-N-methylacridone formed six hydrogen bonds with Leu-192, Phe-151, and Tyr-105 of protein 7jvr. Additionally, these small molecule active compounds interacted with surrounding amino acid residues of the receptor proteins through van der Waals forces [26]. Ligands bind to specific sites on protein receptors through hydrogen bonding or van der Waals interactions, which form the basis of numerous biological processes such as signal transduction, enzymatic catalysis, and hormone activity. These binding sites are typically composed of amino acid residues that provide a structurally and chemically complementary environment, ensuring high specificity and affinity for the correct ligand.

The molecular docking results indicated that these compounds exhibited good docking scores with the core targets and were exclusively present in the pericarp of Wampee, with no such compounds detected in the seeds. Based on these findings, we infer that the presence of these compounds contributes to the greater anticancer activity of the Wampee pericarp compared to the seeds against breast cancer cells. Ligand–receptor interactions play a critical role in determining receptor functionality, as different ligands can bind to distinct receptors or different sites within the same receptor, thereby triggering diverse biological responses. For example, the bioactive compounds from Wampee pericarp may exert their anticancer effects by modulating key signaling pathways through strong interactions with PTGER3, DRD2, and ADORA2A at distinct binding sites. These interactions likely regulate cellular processes involved in tumor growth and proliferation. Therefore, these compounds may be the key active components responsible for the inhibitory effects of Wampee pericarp on breast cancer cells.

### 3.6. Validation Analysis

MDA-MB-231 and MCF-7 cells were used to perform the validation analysis. Cell injury was observed after pericarp extract treatment (Figure S5). In this study, network pharmacology techniques were employed to predict potential targets influenced by pericarp extract, identifying PTGER3, DRD2, and ADORA2A as key targets. It was reported that decreased expression of PTGER3 and DRD2, along with increased expression of ADORA2A, might be associated with an unfavorable prognosis in cancer [27,28,29,30]. Consequently, immunoblotting analysis was conducted to investigate the impact of pericarp extract on the expression levels of PTGER3, DRD2, and ADORA2A. The results demonstrated that pericarp extract upregulated the expression of DRD2 while downregulating the expression of ADORA2A. These findings suggest that pericarp extract induces apoptosis in breast cancer cells by promoting the expression of the anticancer protein DRD2 and suppressing the expression of the pro-cancer protein ADORA2A.

**Figure 7:**
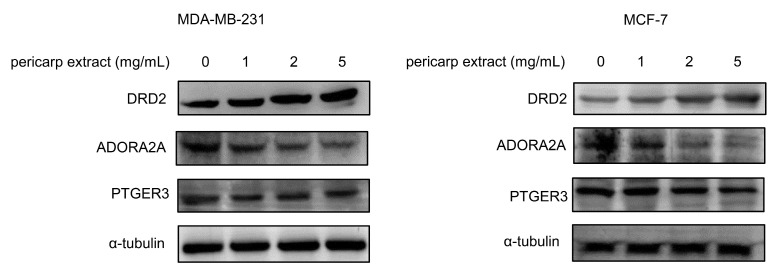
MDA-MB-231 and MCF-7 cells were treated with various concentrations of Wampee pericarp extract (0, 1, 2, 5 mg/mL) for 24 h. A Western blot assay was performed to detect the expression levels of DRD2, PTGER3, and ADORA2A.

Although our experiments did not show a significant regulatory effect of pericarp extract on the protein level of PTGER3, this does not necessarily imply that PTGER3 does not play a role in the anti-tumor activity of pericarp extract (Figure 7). Our molecular docking analysis indicated that compounds such as Wampetin have a strong affinity for PTGER3. This suggests that these compounds may influence PTGER3 activation, potentially enhancing the ability of pericarp extract to suppress the proliferation of breast cancer cells. This finding provides a theoretical foundation for further research into the anticancer mechanisms of Wampee pericarp.

The pericarp of Wampee has been identified as particularly abundant in flavonoids, alkaloids, and coumarins, which are recognized for their diverse biological activities, especially in antioxidant and anti-tumor applications. For example, proanthocyanidins derived from the pericarp have been reported to exhibit significant inhibitory activity against tyrosinase, potentially exerting anti-tumor effects through the chelation of bimetallic copper ions associated with the enzyme [31]. Furthermore, 8-hydroxypsoralen has demonstrated potent anticancer activity and free radical scavenging capabilities in vitro [32]. Nevertheless, the specific anti-tumor active constituents present in Wampee pericarp extract remain to be fully elucidated. Our study has identified several chemical components with anti-tumor activity within this extract, as detailed in Table 1, such as Nootkatone, (+)-ar-Turmerone, and Centauridin, which exhibit significant anti-tumor efficacy. This discovery lays a foundational framework for understanding the mechanisms that underlie the anti-tumor activity of pericarp extract.

## 4. Conclusions

This study underscored the potential application of Wampee pericarp in the treatment of breast cancer. We conducted a comprehensive analysis of the anti-tumor effects of Wampee pericarp, utilizing a multifaceted approach that included metabolomics, network pharmacology, molecular docking, and experimental validation to elucidate the underlying mechanisms. The metabolomics analysis identified several chemical constituents with anti-tumor activity within the pericarp extract, thereby establishing a foundational framework for understanding the mechanisms that contribute to the anti-tumor activity of the extract. Furthermore, the integration of network pharmacology and molecular docking analyses revealed key targets associated with the anti-tumor effects of the pericarp, including PTGER3, DRD2, and ADORA2A, which present novel therapeutic targets for breast cancer treatment. By elucidating the anti-tumor potential of Wampee pericarp and its mechanisms of action, our study provides a theoretical foundation for its application as a functional food ingredient with anticancer properties.

## Figures and Tables

**Table 1:** Structure of active compounds in Wampee pericarp extract.

Compound	Structure	Compound	Structure
2′,3′-Epoxyindicolactone	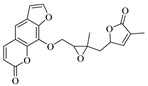	Isorhamnetin	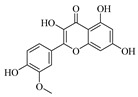
Wampetin	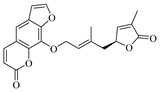	Syringetin	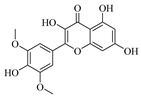
8-geranyloxypsoralen	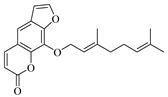	D-Raffinose	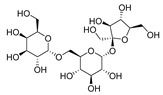
Octyl hydrogen phthalate	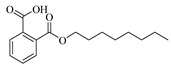	1,2,3,4-Tetrahydro-3-carboxyharmane	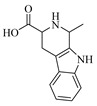
Quercetin	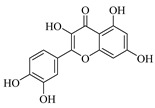	1,3-dihydroxy-N-methylacridone	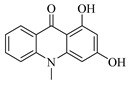
Nootkatone	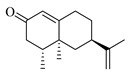	(+)-ar-Turmerone	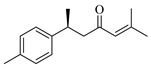
Adenosine	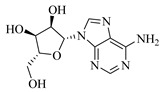	Centaureidin	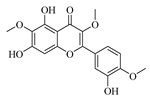
Myricetin	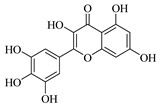		

**Table 2:** Binding affinities of key compounds from Wampee pericarp with target proteins.

Target Proteins	PDB ID	Compound	Binding Energy (kcal/mol)
PTGER3	6m9t	Wampetin	−6.19
DRD2	7jvr	Nootkatone	−7.56
		1,3-dihydroxy-N-methylacridone	−6.99
		(+)-ar-Turmerone	−5.85
		1,2,3,4-Tetrahydro-3-carboxyharmane	−5.73
ADORA2A	5iu4	Myricetin	−5.70
		1,3-dihydroxy-N-methylacridone	−5.50
		2′,3′-Epoxyindicolactone	−5.19

## Data Availability

The original contributions presented in this study are included in the article/Supplementary Materials. Further inquiries can be directed to the corresponding authors.

## Supplementary Materials

The following supporting information can be downloaded at: https://www.mdpi.com/article/10.3390/foods13233878/s1, Table S1: Phytochemicals identified from the pericarp and seeds of Wampee by UPLC-Q-Orbitrap-MS; Table S2: Identification results of GNPS compounds; Figure S1: UPLC-Q-Orbitrap HRMS chromatograms of the pericarp of Wampee in negative ion mode; Figure S2: UPLC-Q-Orbitrap HRMS chromatograms of the pericarp of Wampee in positive ion mode; Figure S3: UPLC-Q-Orbitrap HRMS chromatograms of the seeds of Wampee in negative ion mode; Figure S4: UPLC-Q-Orbitrap HRMS chromatograms of the seeds of Wampee in positive ion mode; Figure S5: Effect of Wampee pericarp on cell morphology in MDA-MB-231 and MCF-7 cells after 24-h treatment.
