# Supplementary material for: Study on the Chemical Composition and Anti-Tumor Mechanisms of Clausena lansium Fruit By-Products: Based on LC-MS, Network Pharmacology Analysis, and Protein Target Validation
Source: Foods. 2024 Nov 30;13(23):3878. doi: 10.3390/foods13233878 (PMC11640417; doi:10.3390/foods13233878)
Supplement: Supplementary file 1 [file foods-13-03878-s001.zip › foods-3349847-supplementary.pdf]

Study on the Chemical Composition and Anti-Tumor Mechanisms of *Clausena lansium* Fruit By-Products: Based on LC-MS, Network Pharmacology Analysis, and Protein Target Validation

Ziyue Zhang [1,2,†], Liangqian Zhang [3,†], Pengfei Wu [2,†], Yuan Tian [3], Yao Wen [4], Meina Xu [2], Peihao Xu [1,5], Ying Jiang [2], Nan Ma [2,*], Qi Wang [3] and Wei Dai [1,5,*]

1. Teaching and Experimental Center, Guangdong Pharmaceutical University, Guangzhou 510006, China
2. College of Pharmacy, Jinan University, Guangzhou 510632, China
3. Key Laboratory of Xinjiang Phytomedicine Resource and Utilization, Ministry of Education, School of Pharmacy, Shihezi University, Shihezi 832002, China
4. School of Pharmacy, Guangdong Pharmaceutical University, Guangzhou 510006, China
5. Comprehensive Experimental Teaching Center of Traditional Chinese Medicine, Yunfu Campus, Guangdong Pharmaceutical University, Yunfu 527500, China

* Correspondence: nanma927@126.com (N.M.); dai_gdpu_2018@gdpu.edu.cn (W.D.)
† These authors contributed equally to this work.

Table S1. Phytochemicals identified from the pericarp and seeds of Wampee by UPLC-Q-Orbitrap-MS
Table S2. Identification results of GNPS compounds
Figure S1. UPLC-Q-Orbitrap HRMS chromatograms of the pericarp of Wampee in negative ion mode
Figure S2. UPLC-Q-Orbitrap HRMS chromatograms of the pericarp of Wampee in positive ion mode
Figure S3. UPLC-Q-Orbitrap HRMS chromatograms of the seeds of Wampee in negative ion mode
Figure S4. UPLC-Q-Orbitrap HRMS chromatograms of the seeds of Wampee in positive ion mode
Figure S5. Effect of Wampee pericarp on cell morphology in MDA-MB-231 and MCF-7 cells after 24-hour treatment

Table S1. Phytochemicals identified from the pericarp and seeds of Wampee by UPLC-Q-Orbitrap-MS

No	Name	Formula	Error [ppm]	RT [min]		m/z	Reference Ion	Classification
				pericarp	seeds			
1	4-Aminobutanoic acid	$C_4H_9NO_2$	0.12		0.718	104.07062	$[M+H]^+$	Alkaloids
2	Proline	$C_5H_9NO_2$	-0.62		0.742	116.07053	$[M+H]^+$	Alkaloids
3	Betaine	$C_5H_{11}NO_2$	-0.20	0.78		118.08624	$[M+H]^+$	Alkaloids
4	Pipecolic acid	$C_6H_{11}NO_2$	0.49	0.788	0.763	130.08632	$[M+H]^+$	Alkaloids
5	L-Iditol	$C_6H_{14}O_6$	-0.33	0.795	0.795	181.0717	$[M-H]^-$	Alcohols
6	Sucrose	$C_{12}H_{22}O_{11}$	-1.40		0.795	341.10834	$[M-H]^-$	Sugars
7	D-(+)-Mannose	$C_6H_{12}O_6$	-0.34	0.797		179.05606	$[M-H]^-$	Sugars
8	D-Raffinose	$C_{18}H_{32}O_{16}$	-0.75	0.809	0.81	549.16693	$[M+FA-H]^-$	Sugars
9	Galactonic acid	$C_6H_{12}O_7$	-1.79	0.813		195.05067	$[M-H]^-$	Organic acids
10	Malonic acid	$C_3H_4O_4$	-0.70	0.827	0.898	103.00361	$[M-H]^-$	Organic acids
11	L-Threonic acid	$C_4H_8O_5$	-0.71		0.832	135.0298	$[M-H]^-$	Organic acids
12	D-(-)-Quinic acid	$C_7H_{12}O_6$	-1.06		0.842	191.05591	$[M-H]^-$	Organic acids
13	trans-Aconitic acid	$C_6H_6O_6$	-1.12	0.863		173.00896	$[M-H]^-$	Organic acids
14	DL-Malic acid	$C_4H_6O_5$	-0.85		0.876	133.01413	$[M-H]^-$	Organic acids
15	Adenosine	$C_{10}H_{13}N_5O_4$	0.24	0.987	1.009	268.1041	$[M+H]^+$	Alkaloids
16	Nicotinic acid	$C_6H_5NO_2$	0.10		1.009	124.0393	$[M+H]^+$	Alkaloids
17	2-Hydroxyphenylalanine	$C_9H_{11}NO_3$	-0.12		1.028	182.08115	$[M+H]^+$	Phenols
18	Guanosine	$C_{10}H_{13}N_5O_5$	-0.13		1.035	284.09891	$[M+H]^+$	Alkaloids
19	D-(+)-Malic acid	$C_4H_6O_5$	-0.82		1.109	133.01413	$[M-H]^-$	Ethers
20	Mesaconic acid	$C_5H_6O_4$	-1.42	1.112		111.00858	$[M-H-H_2O]^-$	Organic acids
21	cis-Aconitic acid	$C_6H_6O_6$	-1.33	1.112		173.00893	$[M-H]^-$	Organic acids
22	Itaconic acid	$C_5H_6O_4$	-0.59		1.124	111.0087	$[M-H-H_2O]^-$	Organic acids

23	Citric acid	$C_6H_8O_7$	0.10		1.126	191.01974	$[M-H]^-$	Organic acids
24	Fumaric acid	$C_4H_4O_4$	-0.96		1.178	115.00357	$[M-H]^-$	Organic acids
25	Gluconic acid	$C_6H_{12}O_7$	-0.55	1.209		195.05092	$[M-H]^-$	Organic acids
26	Isoleucine	$C_6H_{13}NO_2$	-0.05		1.215	132.1019	$[M+H]^+$	Alkaloids
27	Kojic acid	$C_6H_6O_4$	-0.40	1.226		143.03383	$[M+H]^+$	Esters
28	δ-Ribono-1,4-lactone	$C_5H_8O_5$	-0.96	1.227		147.02975	$[M-H]^-$	Esters
29	Succinic acid	$C_4H_6O_4$	-1.03	1.232	1.243	117.01921	$[M-H]^-$	Organic acids
30	Meglutol	$C_6H_{10}O_5$	-0.84	1.278	1.298	161.04541	$[M-H]^-$	Organic acids
31	Gallic acid	$C_7H_6O_5$	-0.51	1.385		169.01416	$[M-H]^-$	Phenols
32	methyl citrate	$C_7H_{10}O_7$	-0.40	1.544		205.03528	$[M-H]^-$	Organic acids
33	Epigallocatechin	$C_{15}H_{14}O_7$	-0.55	2.174		305.06644	$[M-H]^-$	Phenols
34	Catechol	$C_6H_6O_2$	-0.83	2.571		109.02941	$[M-H]^-$	Phenols
35	Gentisic acid	$C_7H_6O_4$	-0.89	2.575		153.0192	$[M-H]^-$	Phenols
36	Indole-3-acrylic acid	$C_{11}H_9NO_2$	-0.37		3.137	188.07053	$[M+H]^+$	Alkaloids
37	DL-4-Hydroxyphenyllactic acid	$C_9H_{10}O_4$	-0.79	3.31		181.05049	$[M-H]^-$	Phenylpropanoids
38	1,2,3,4-Tetrahydro-3-carboxyharmane	$C_{13}H_{14}N_2O_2$	0.02	5.363		231.11281	$[M+H]^+$	Alkaloids
39	Quercetin 3-O-β-galactopyranosyl-(1→4)-α-rhamnopyranoside-7-O-β-galactopyranoside	$C_{33}H_{40}O_{21}$	-0.37	5.371		771.19849	$[M-H]^-$	Flavonoids
40	3-Coumaric acid	$C_9H_8O_3$	-0.27		6.553	163.04002	$[M-H]^-$	Phenylpropanoids
41	Manghaslin	$C_{33}H_{40}O_{20}$	-0.48	6.627		755.20349	$[M-H]^-$	Flavonoids
42	Scopoletin	$C_{10}H_8O_4$	0.05		6.683	193.04955	$[M+H]^+$	Coumarins
43	Myricetin 3-galactoside	$C_{21}H_{20}O_{13}$	-0.43	6.761		479.08286	$[M-H]^-$	Flavonoids
44	Rutin	$C_{27}H_{30}O_{16}$	-0.73	7.635	7.638	609.14551	$[M-H]^-$	Flavonoids
45	Myricitrin	$C_{21}H_{20}O_{12}$	-0.52	7.648		463.08792	$[M-H]^-$	Flavonoids

46	Myricetin	$C_{15}H_{10}O_8$	0.05	7.661		319.04486	$[M+H]^+$	Flavonoids
47	N-Acetyl-DL-tryptophan	$C_{13}H_{14}N_2O_3$	-0.73		7.933	245.09299	$[M-H]^-$	Alkaloids
48	Kaempferol-3-O-rutinoside	$C_{27}H_{30}O_{15}$	-0.73		8.561	593.15076	$[M-H]^-$	Flavonoids
49	Kaempferol-7-O-neohesperidoside	$C_{27}H_{30}O_{15}$	-0.12	8.569		593.15106	$[M-H]^-$	Flavonoids
50	Isorhamnetin 3-O-neohesperidoside	$C_{28}H_{32}O_{16}$	-0.51		8.773	623.16144	$[M-H]^-$	Flavonoids
51	Purifolinin	$C_{22}H_{22}O_{12}$	-0.19	8.947		477.10376	$[M-H]^-$	Flavonoids
52	Quercetin 3-methyl ether 3'-xyloside	$C_{21}H_{20}O_{11}$	-0.60	8.986	9.000	447.09302	$[M-H]^-$	Flavonoids
53	Syringetin-3-O-galactoside	$C_{23}H_{24}O_{13}$	-0.67	9.119		507.11407	$[M-H]^-$	Flavonoids
54	Quercitrin	$C_{21}H_{20}O_{11}$	0.16	9.161		449.10791	$[M+H]^+$	Flavonoids
55	5,8-Dihydroxy-2-(4-hydroxyphenyl)-7-methoxy-4-oxo-4H-chromen-3-yl 6-deoxy-α-L-mannopyranoside	$C_{22}H_{22}O_{11}$	-0.23		9.475	463.12338	$[M+H]^+$	Flavonoids
56	Syringetin-3-glucoside	$C_{23}H_{24}O_{13}$	0.03	9.649		509.129	$[M+H]^+$	Flavonoids
57	Isorhamnetin-3-O-rutinoside	$C_{28}H_{32}O_{16}$	-0.41	9.882		623.1615	$[M-H]^-$	Flavonoids
58	Isorhamnetin-3-O-nehesperidine	$C_{28}H_{32}O_{16}$	0.02	10.067		625.17633	$[M+H]^+$	Flavonoids
59	Isorhamnetin	$C_{16}H_{12}O_7$	0.20	10.463	10.270	317.06564	$[M+H]^+$	Flavonoids
60	Quercetin	$C_{15}H_{10}O_7$	-0.22	11.655		301.03531	$[M-H]^-$	Flavonoids
61	N-[2-(4-Methoxyphenyl)ethyl]-3-methyl-2-butenamide	$C_{14}H_{19}NO_2$	-0.16		13.417	234.14882	$[M+H]^+$	Alkaloids
62	Syringetin	$C_{17}H_{14}O_8$	-0.28	13.713		345.06149	$[M-H]^-$	Flavonoids
63	3H-Benzo[e]indole-2-carboxylic acid	$C_{13}H_9NO_2$	-0.01	14.211		212.0706	$[M+H]^+$	Alkaloids
64	Oxypeucedanin	$C_{16}H_{14}O_5$	0.29		14.604	287.09143	$[M+H]^+$	Coumarins
65	Centaureidin	$C_{18}H_{16}O_8$	0.26	16.067		361.09189	$[M+H]^+$	Flavonoids
66	Clausenacoumarine	$C_{21}H_{18}O_6$	-0.31		16.681	367.11746	$[M+H]^+$	Coumarins
67	1,3-dihydroxy-N-methyl acridone	$C_{14}H_{11}NO_3$	-0.30	16.729		240.06654	$[M-H]^-$	Alkaloids

68	2',3'-Epoxyindicolactone	$C_{21}H_{18}O_7$	0.03	16.735		383.11255	$[M+H]^+$	Flavonoids
69	(E)-N-phenethyl-3-phenyl-acrylamide	$C_{17}H_{17}NO$	-0.42		16.743	252.13818	$[M+H]^+$	Alkaloids
70	Lauryldimethylamine oxide	$C_{14}H_{31}NO$	-0.17		17.256	230.2478	$[M+H]^+$	Alkaloids
71	4-Ethoxy ethylbenzoate	$C_{11}H_{14}O_3$	-0.36		18.121	195.1015	$[M+H]^+$	Esters
72	Triallyl Cyanurate	$C_{12}H_{15}N_3O_3$	0.08	19.650		250.11864	$[M+H]^+$	Alkaloids
73	Asperglaucide	$C_{27}H_{28}N_2O_4$	0.14	19.652	19.360	445.21225	$[M+H]^+$	Alkaloids
74	1,3:2,4-Bis(p-ethylbenzylidene)sorbitol	$C_{24}H_{30}O_6$	0.13	20.044		415.21152	$[M+H]^+$	Ethers
75	Polygodial	$C_{15}H_{22}O_2$	0.30	21.173		235.16933	$[M+H]^+$	Aldehydes
76	2-Amino-1,3,4-octadecanetriol	$C_{18}H_{39}NO_3$	0.07	20.630	20.630	318.30029	$[M+H]^+$	Alkaloids
77	Linoleoyl ethanolamide	$C_{20}H_{37}NO_2$	-0.10		21.76	324.28967	$[M+H]^+$	Alkaloids
78	Asperphenamate	$C_{32}H_{30}N_2O_4$	-0.18	21.773	21.110	507.22772	$[M+H]^+$	Alkaloids
79	trans-Anethole	$C_{10}H_{12}O$	-0.05	21.957		149.09608	$[M+H]^+$	Phenylpropanoids
80	1-Linolenoylglycerol	$C_{21}H_{36}O_4$	0.12	22.064		353.26868	$[M+H]^+$	Esters
81	(+)-ar-Turmerone	$C_{15}H_{20}O$	-0.28	22.54		217.15863	$[M+H]^+$	Terpenoids
82	(-)-Caryophyllene oxide	$C_{15}H_{24}O$	-0.30	22.873		221.18993	$[M+H]^+$	Terpenoids
83	4-Methoxycinnamic acid	$C_{10}H_{10}O_3$	-0.62		23.156	179.07016	$[M+H]^+$	Phenylpropanoids
84	Octyl hydrogen phthalate	$C_{16}H_{22}O_4$	0	23.411		279.15909	$[M+H]^+$	Esters
85	(1S)-Tricyclo[7.3.1.$0^{2,7}$]tridec-2(7)-en-13-on	$C_{13}H_{18}O$	-0.36		23.966	191.14293	$[M+H]^+$	Terpenoids
86	Pheophorbide A	$C_{35}H_{36}N_4O_5$	-1.25		23.99	593.27509	$[M+H]^+$	Alkaloids
87	N-(14-Methylpentadecanoyl)phenylalanine	$C_{25}H_{41}NO_3$	-0.01		24.251	404.31592	$[M+H]^+$	Alkaloids
88	2,2'-Methylenebis[6-tert-butyl-p-cresol]	$C_{23}H_{32}O_2$	-1.65	25.12	25.117	339.23239	$[M-H]^-$	Phenols
89	8-Geranyloxypsoralen	$C_{21}H_{22}O_4$	-0.21	23.040	23.040	339.159	$[M+H]^+$	Coumarins
90	Nootkatone	$C_{15}H_{22}O$	-0.05	25.68		219.17433	$[M+H]^+$	Terpenoids
91	Bergamottin	$C_{21}H_{22}O_4$	-0.27	26.057		339.159	$[M+H]^+$	Coumarins

92	Hexadecanamide	$C_{16}H_{33}NO$	-0.25	25.090	25.090	256.26343	$[M+H]^+$	Alkaloids
93	L-α-Palmitin	$C_{19}H_{38}O_4$	0.14	25.690	25.680	331.28433	$[M+H]^+$	Esters
94	Stearic Acid	$C_{18}H_{36}O_2$	0.32	27.981		283.26434	$[M-H]^-$	Organic acids
95	Stearoyl ethanolamide	$C_{20}H_{41}NO_2$	0.21	26.190	26.180	328.32108	$[M+H]^+$	Alkaloids
96	Oleoyl ethanolamide	$C_{20}H_{39}NO_2$	0.10	28.268		326.30539	$[M+H]^+$	Alkaloids
97	Stearamide	$C_{18}H_{37}NO$	-0.94	26.850	26.850	284.29453	$[M+H]^+$	Alkaloids
98	Lignoceric Acid	$C_{24}H_{48}O_2$	-0.33	28.33	28.331	367.35803	$[M-H]^-$	Organic acids
99	Tridemorph	$C_{19}H_{39}NO$	0.15	28.332		298.31049	$[M+H]^+$	Alkaloids
100	Diisooctyl phthalate	$C_{24}H_{38}O_4$	0.17	27.830	27.850	391.28436	$[M+H]^+$	Esters
101	Erucamide	$C_{22}H_{43}NO$	-0.47	28.816	28.280	338.34158	$[M+H]^+$	Alkaloids
102	Docosanamide	$C_{22}H_{45}NO$	0.09	28.851		340.35742	$[M+H]^+$	Alkaloids
103	Xanthoxol	$C_{11}H_6O_4$	-0.12	18.05		203.03377	$[M+H]^+$	Coumarins
104	Wampetin	$C_{21}H_{18}O_6$	-0.22	18.86		367.11743	$[M+H]^+$	Coumarins
105	Lansamide I	$C_{18}H_{17}NO$	-0.65		20.01	264.13812	$[M+H]^+$	Alkaloids
106	Lansiumamide C	$C_{18}H_{19}NO$	-0.72		19.23	266.15375	$[M+H]^+$	Alkaloids
107	Homoclausenamide	$C_{18}H_{17}NO_2$	-0.63		18.70	280.13303	$[M+H]^+$	Alkaloids

Table S2 Identification results of GNPS compounds

NO	Name	Formula	RT		Cosine	Spec MZ	Lib MZ	Classification	Ion mode	Source	
			pericarp	seeds						pericarp	seeds
1	Azacyclotridecan-2-one	$C_{12}H_{23}NO$	26.15	26.15	0.99	198.19	198.19	Alkaloids	Positive ion mode	+	+
2	Bisabolol	$C_{15}H_{26}O$	23.03	23.03	0.98	205.19	205.19	Terpenoids	Positive ion mode	+	+
3	Adenosine	$C_{10}H_{13}N_5O_4$	1.11	1.12	0.97	268.10	267.24	Alkaloids	Positive ion mode	+	+
4	Tyrosine	$C_9H_{11}NO_3$	-	19.23	0.96	182.08	182.08	Alkaloids	Positive ion mode	-	+
5	D-Glucono-1,5-lactone	$C_6H_{10}O_6$	16.11	-	0.93	179.06	179.06	Sugars	Positive ion mode	+	-
6	1-Monopalmitin	$C_{19}H_{38}O_4$	25.68	25.68	0.91	331.28	331.28	Esters	Positive ion mode	+	+
7	Myristamidopropyl betaine	$C_{21}H_{42}N_2O_3$	20.17	20.17	0.89	371.33	371.33	Alkaloids	Positive ion mode	+	+
8	Linoleoyl ethanolamide	$C_{20}H_{37}NO_2$	23.55	23.55	0.85	324.29	324.29	Organic acids	Positive ion mode	+	+
9	Palmitic acid	$C_{16}H_{32}O_2$	25.70	25.70	0.84	239.24	239.24	Organic acids	Positive ion mode	+	+
10	Sucrose	$C_{12}H_{22}O_{11}$	0.81	0.81	0.83	360.15	360.15	Sugars	Positive ion mode	+	+
11	Lauryldiethanolamine	$C_{16}H_{35}N$	18.58	18.58	0.76	274.27	274.27	Organic	Positive	+	+

		O_2						acids	ion mode		
12	8-Geranyloxypsoralen	$C_{21}H_{22}O_4$	23.04	23.04	0.74	339.16	339.16	Coumarins	Positive ion mode	+	+
13	(+)-Nootkatone	$C_{15}H_{22}O$	27.14	-	0.73	201.16	201.16	Terpenoids	Positive ion mode	+	-
14	1-beta-D-Glucopyranosyl-L-tryptophan	$C_{17}H_{22}N_2O_7$	-	3.07	0.73	367.15	367.15	Alkaloids	Positive ion mode	-	+
15	1-Methyl-4-(6-methyl-5-hepten-2-yl)-2,3-dioxabicyclo[2.2.2]oct-5-en	$C_{15}H_{24}O_2$	22.45	-	0.72	219.17	219.17	Terpenoids	Positive ion mode	+	-
16	Aurantiamide acetate	$C_{27}H_{28}N_2O_4$	21.773	21.110	0.71	445.21	445.21	Alkaloids	Positive ion mode	+	+
17	Kaempferol 3-O-rutinoside	$C_{27}H_{30}O_{15}$	8.56	8.56	0.7	595.17	595.17	Flavonoids	Positive ion mode	+	+
18	Lauramidopropylbetaine	$C_{19}H_{39}N_2O_3+$	20.09	20.09	0.7	343.3	343.3	Alkaloids	Positive ion mode	+	+
19	D-(-)-Mannitol	$C_6H_{14}O_6$	0.80	0.80	0.89	181.07	181.07	Sugars	Negative ion mode	+	+
20	Quercetin	$C_{15}H_{10}O_7$	11.66	-	0.89	301.04	301.04	Flavonoids	Negative ion mode	+	-

+: It represents containing the compound; -: It represents does not contain the compound

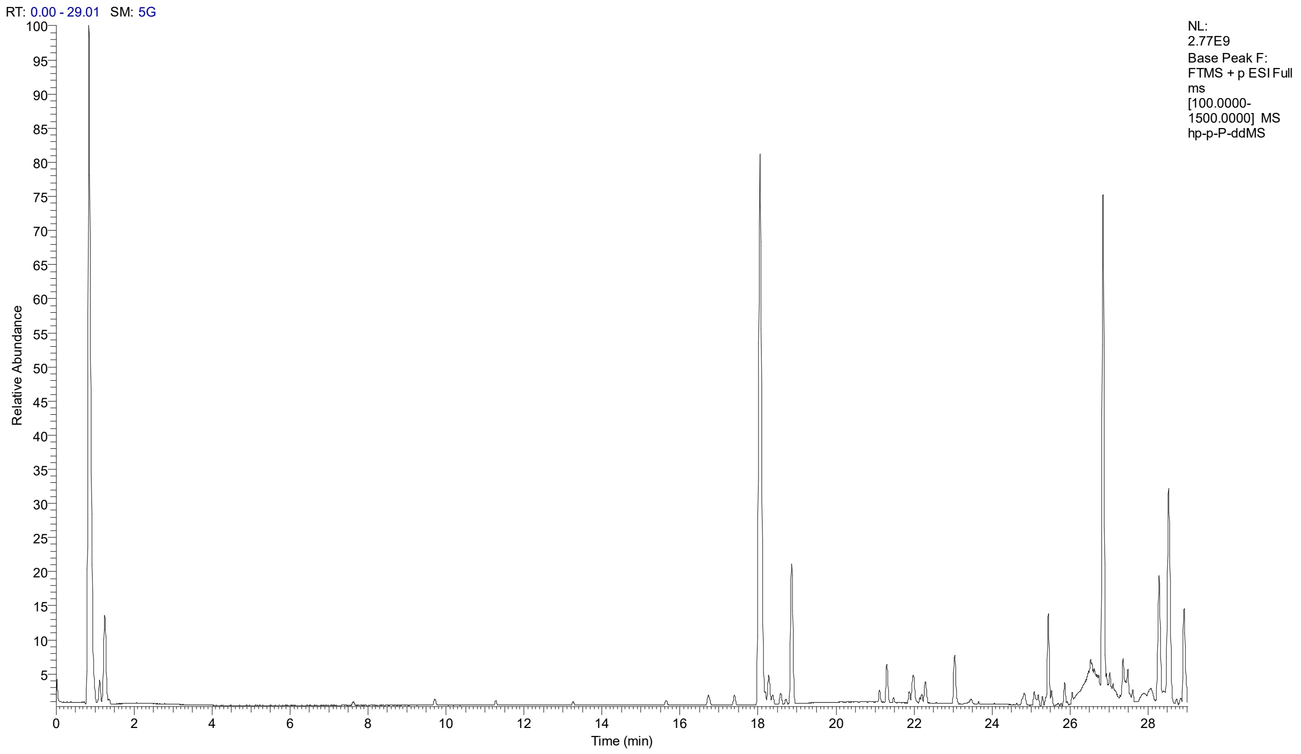

Figure S1. UPLC-Q-Orbitrap HRMS chromatograms of the pericarp of Wampee in positive ion mode

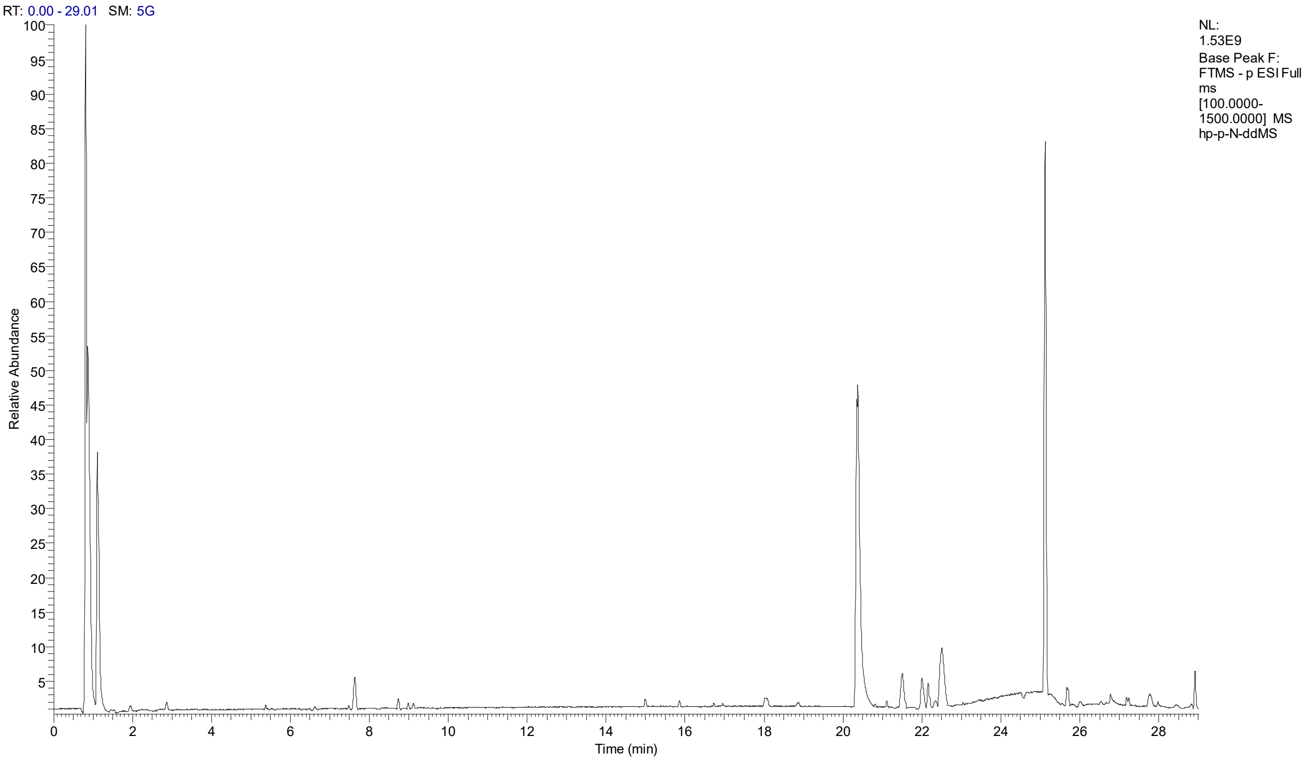

Figure S2. UPLC-Q-Orbitrap HRMS chromatograms of the pericarp of Wampee in negative ion mode

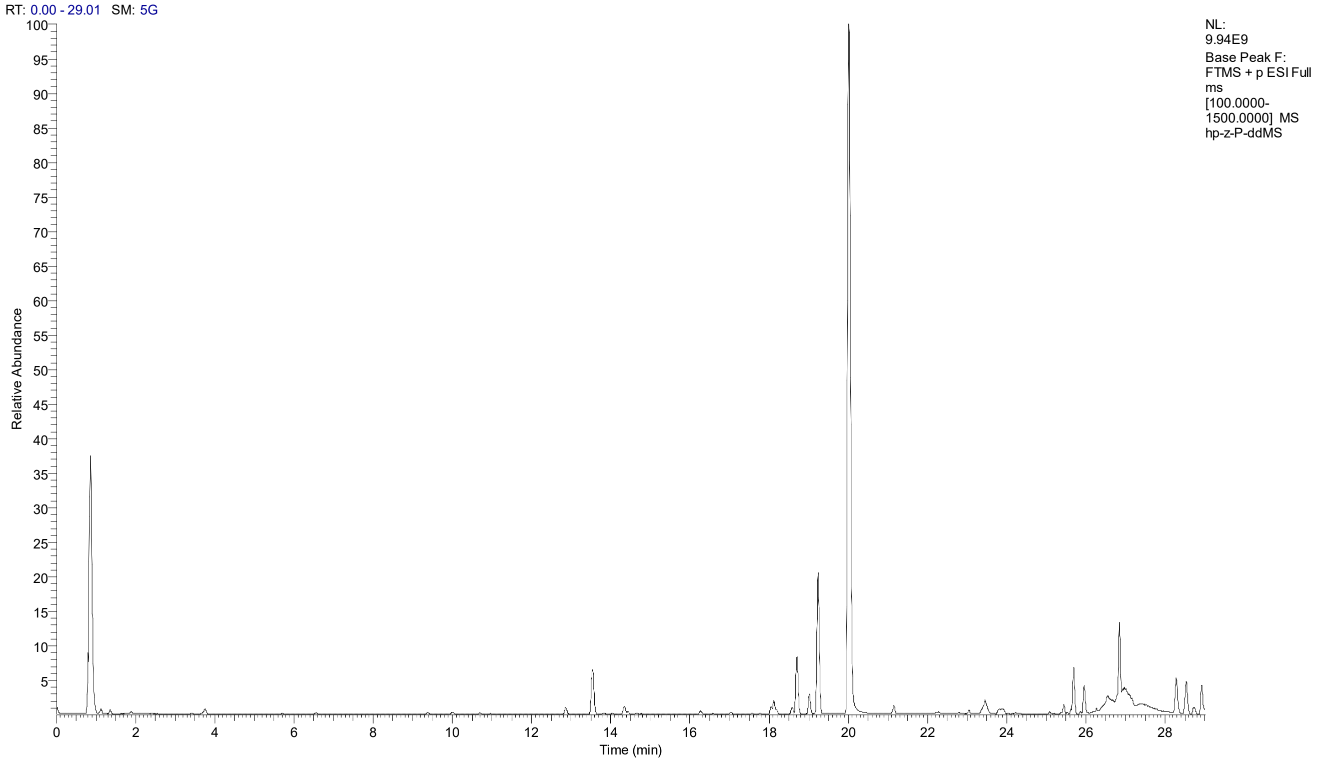

Figure S3. UPLC-Q-Orbitrap HRMS chromatograms of the seeds of Wampee in positive ion mode

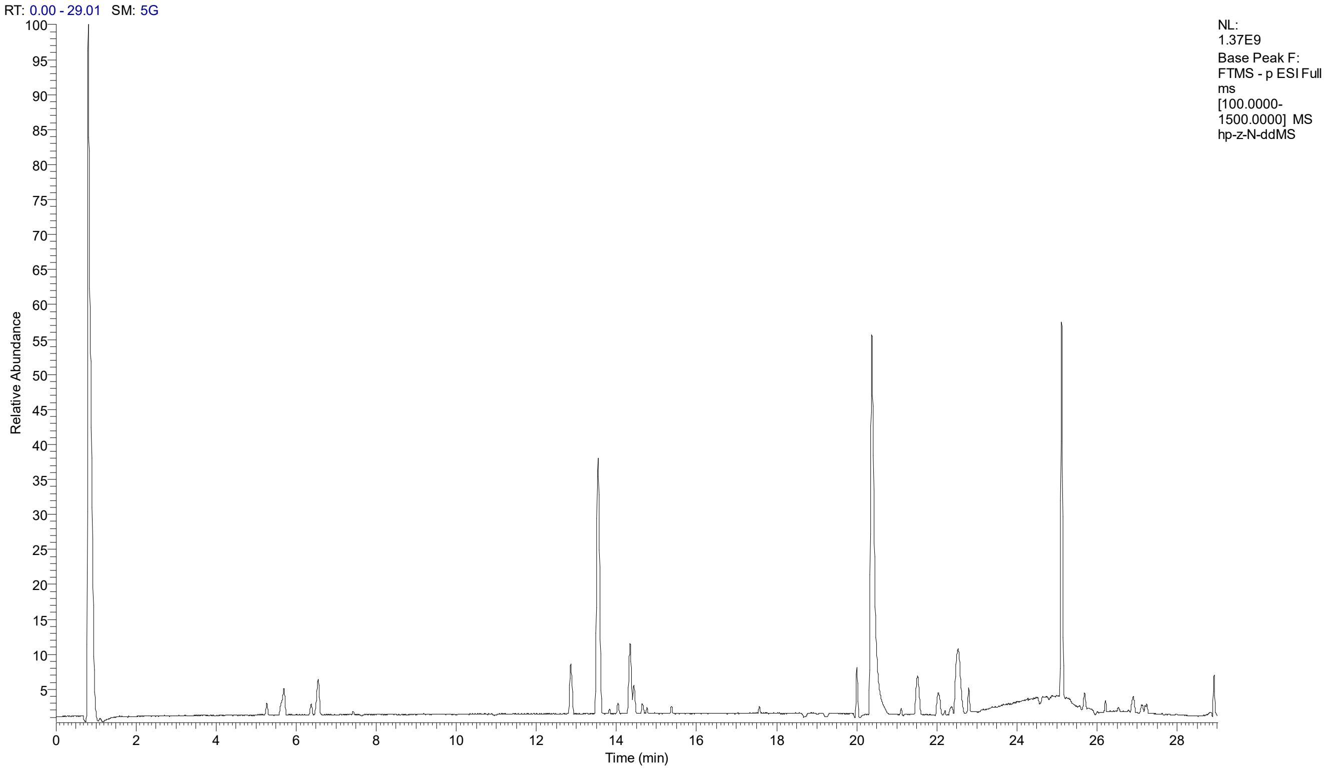

Figure S4. UPLC-Q-Orbitrap HRMS chromatograms of the seeds of Wampee in negative ion mode

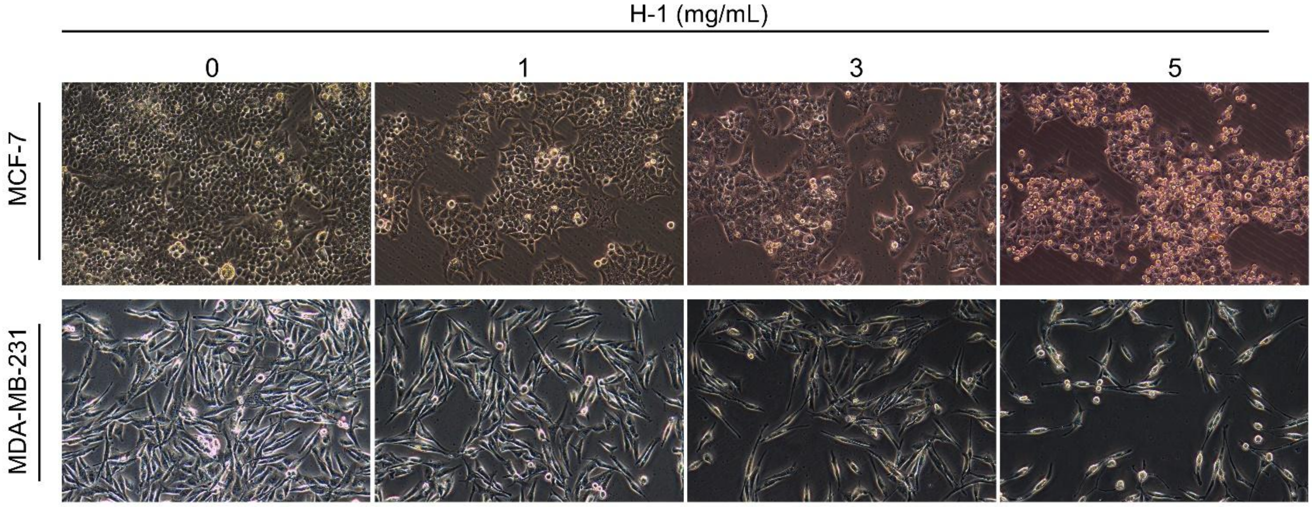

Figure S5. Effect of Wampee pericarp on cell morphology in MDA-MB-231 and MCF-7 cells after 24-hour treatment
